# The recurrence of colonic volvulus due to nonrotation after intestinal resection in adulthood: a case report

**DOI:** 10.1186/s40792-019-0710-x

**Published:** 2019-10-21

**Authors:** Yusuke Sakimura, Hirotaka Kitamura, Noriyuki Inaki, Hiroyuki Bando

**Affiliations:** 1 Department of Gastroenterological Surgery, Ishikawa Prefectural Central Hospital, 2-1 Kuratuki Higashi, Kanazawa, Ishikawa 9208530 Japan; 2 Department of Surgery, Juntendo Urayasu Hospital, Juntendo University, 2-1-1, Tomioka, Urayasu-shi, Chiba 2790021 Japan

**Keywords:** Intestinal volvulus, Intestinal rotational disorder, Nonrotation, Emergency surgery

## Abstract

**Background:**

Intestinal nonrotation is a rare congenital condition that causes fatal colonic volvulus at any age. Once volvulus attack occurs, radical surgical therapy is required for treatment and the prevention of recurrence. This report describes the case of an adult female patient with a recurrence of cecum volvulus due to intestinal nonrotation after transverse colon resection for colonic volvulus.

**Case presentation:**

A 27-year-old female visited our emergency room (ER) with intermittent abdominal pain and nausea. Enhanced computed tomography (CT) showed enlargement of the level of the ascending and transverse colon and an obstruction with a whirlpool sign at the transverse colon. The small intestine was distributed on the right side of the abdominal cavity, and the large intestine occupied the left side. She was diagnosed with volvulus with intestinal nonrotation, and emergency surgery was performed. Surgical examination indicated that the ascending colon to the transverse colon was not fixed to the retroperitoneum, and the transverse colon was rotated 180° clockwise. The axis of the volvulus was a mesenteric adhesion of the transverse colon. The involved transverse colon was resected, and the intestine was reconstructed by functional end-to-end anastomosis (FEEA). Six years after the initial surgery, the patient presented to the ER with abdominal fullness and lower abdominal pain. Enhanced CT revealed that the cecum, ascending colon, and remaining transverse colon were dilated with an obstruction. The appendix was located in the left upper abdominal cavity. The clinical diagnosis was cecal volvulus with intestinal nonrotation. An emergency laparotomy revealed that the cecum was rotated 180° clockwise. The terminal ileum to the remaining transverse colon was resected, and FEEA was performed. Seven months later, she suffered obstruction of the intestine caused by an operative adhesion, and conservative treatment was successful. The patient has had no abdominal symptoms for one and a half years so far.

**Conclusions:**

Surgeons should realize that nonrotation of the intestines induces volvulus in adulthood and should familiarize themselves with its clinical findings, appropriate treatment, and prognosis. Even after surgical treatment, awareness of the recurrence of volvulus should be maintained to avoid a late diagnosis.

## Background

Colonic volvulus can cause bowel obstruction and lead to ischemia, gangrene, and perforation of the involved segment, resulting in death [1]. Various diseases and conditions can induce volvulus of the large intestine, such as pregnancy, previous pelvic surgery, neuropsychological impairment, diabetes, chronic constipation, and institutionalization [1–3]. Anomalies of intestinal rotation during the fetal period are crucial factors for the presentation of volvulus at any age [4–6]. The rate of adult patients varies among reports. Forty-eight percent of patients with this malrotation are diagnosed at an age over 18, either symptomatically or asymptomatically [7], but another study mentioned that approximately 75% of patients are diagnosed before 5 years of age [8]. The anomaly can provoke colonic volvulus in any segment of the large intestine at adulthood, and emergency surgery is needed. However, the prevalence and favored site of the colonic volvulus in adulthood is unclear, with limited case reports [7, 9–14], and no case of recurrence at different segments has been reported. Every surgeon should know that an anomaly of the intestinal rotation is not only a pediatric disease but that adult patients can also be affected [15]. Here, we report a rare case of recurrence of cecal volvulus after resection of the transverse colon for volvulus in a female patient with undiagnosed nonrotation.

## Case presentation

### First surgery

**Fig. 1:**
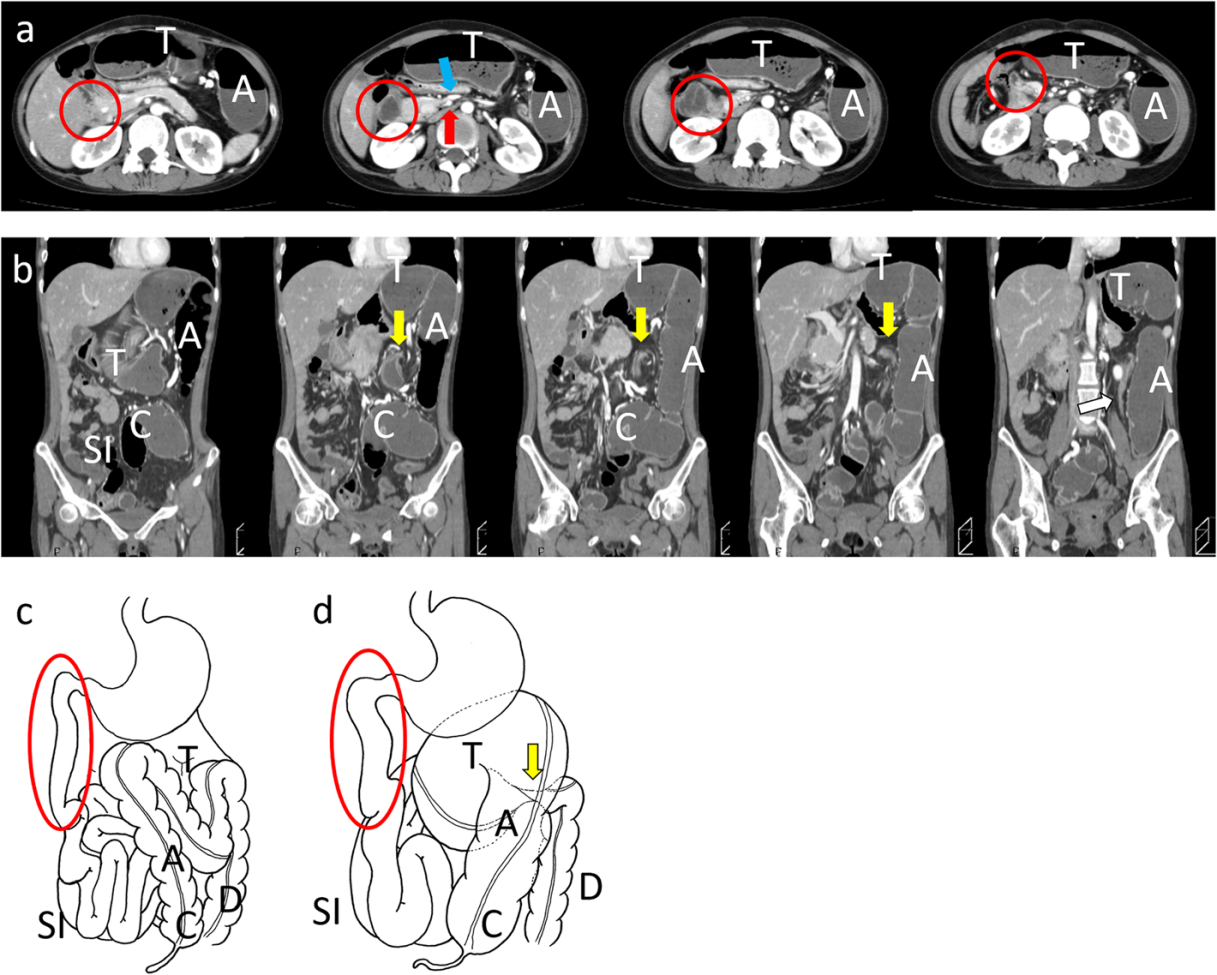
Enhanced computed tomography before the first surgery. Transverse section (**a**) and coronary section (**b**) images show the enlarged transverse colon (T) with an obstructed point at the splenic flexure with a whirlpool sign (yellow arrow). The ascending colon (A), transverse colon (T), and descending colon (D) were located on the left side of the abdominal cavity, and the small intestine (SI) was located on the other side. The cecum (C) inhabited the pelvic cavity. The superior mesenteric artery (red arrow) was located on the right side of the superior mesenteric vein (blue arrow). The duodenum did not cross the vertebral column but traveled down straight (red circle). The drainage veins were dilated, but there were no signs of ischemia. **c** and **d** illustrate the gastrointestinal tract of nonrotation and volvulus at the transverse colon before surgery

A 27-year-old female visited our emergency room (ER) with intermittent abdominal pain and nausea. Her last bowel movement was 2 days before visiting. She had no notable past medical history, including no abdominal operation or growth abnormalities. On her physical examination, she was tachycardic, with a pulse of 136 bpm, but the rest of her vital signs were normal. Abdominal examination revealed distension and epigastric tenderness with peritoneal irritation symptoms. Her laboratory examination results showed slightly elevated C-reactive protein levels (1.8 mg/dL), but the other findings were within normal ranges. Enhanced computed tomography (CT) showed enlargement at the level of the ascending and transverse colon (Fig. 1). The transverse colon had an obstructive point with a whirlpool sign. The colonic wall showed no ischemic signs, but the supply vein was dilated. The large intestine was located on the left side of the abdominal cavity, and the small intestine occupied the other side. The duodenum did not cross between the superior mesenteric artery (SMA) and the abdominal aorta but went down straight on the right side of the SMA. The SMA and the superior mesenteric vein (SMV) were located at inverted positions; the SMA was on the right side, and the SMV was on the left. The patient was diagnosed with transverse colonic volvulus, and nonrotation of the intestine was suspected.

**Fig. 2:**
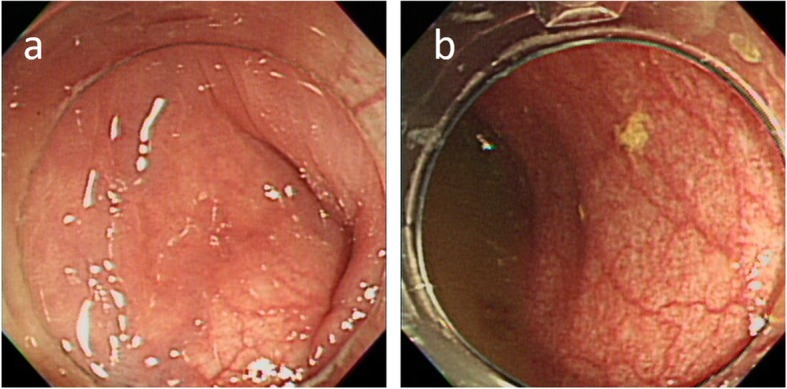
Endoscopic findings before the first surgery. There was an obstructed area of the transverse colon (**a**), and the scope managed to pass through the lesion. The oral side of the colon was dilated and contained with feces. The wall was conjugated and swollen, but no ischemic change was observed (**b**)

**Fig. 3:**
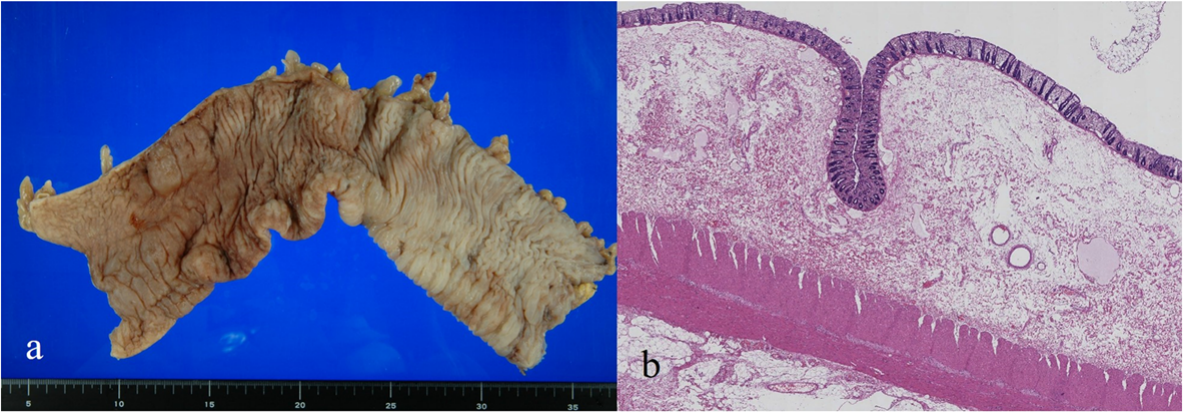
Pathological findings of the first surgery. The enlarged transverse colon was resected (**a**) and showed edema macroscopically. Pathological findings (**b**) revealed edema and conjugated submucosal layers, and the mucosal layer showed hemorrhage and detachment. These findings were inconsistent with the acute circulating disturbance

A transanal ileus tube was placed endoscopically at the dilated colon to decompress the enlargement. The mucosa of the colon was congestive, but there were no necrotic findings (Fig. 2). She was treated conservatively and observed for the next 24 h. However, her symptoms remained, and CT revealed no improvement in the intestinal obstruction and showed increasing ascites. Conservative therapy was determined to be ineffective; therefore, emergency surgery was performed. The laparoscopic view revealed the enlarged transverse colon, and there was no space for the surgical procedure. Consequently, the operation was converted to laparotomy with a middle abdominal incision. The dilated transverse colon was pulled out from the abdominal cavity without any mobilization, and it was rotated with 180° clockwise along a bridging adhesion within the transverse colon itself. The dilated transverse colon was resected (Fig. 3) and reconstructed with functional end-to-end anastomosis (FEEA). The surgical observation also showed that the mesenteries of the ascending colon to the transverse colon had mobility without fixation to the retroperitoneum. There was no ligament formation requiring Ladd’s procedure. The surgical diagnosis was transverse colonic volvulus, nonrotation type, due to the anomaly of bowel rotation. She was discharged uneventfully 12 days after the surgery.

### Second surgery

**Fig. 4:**
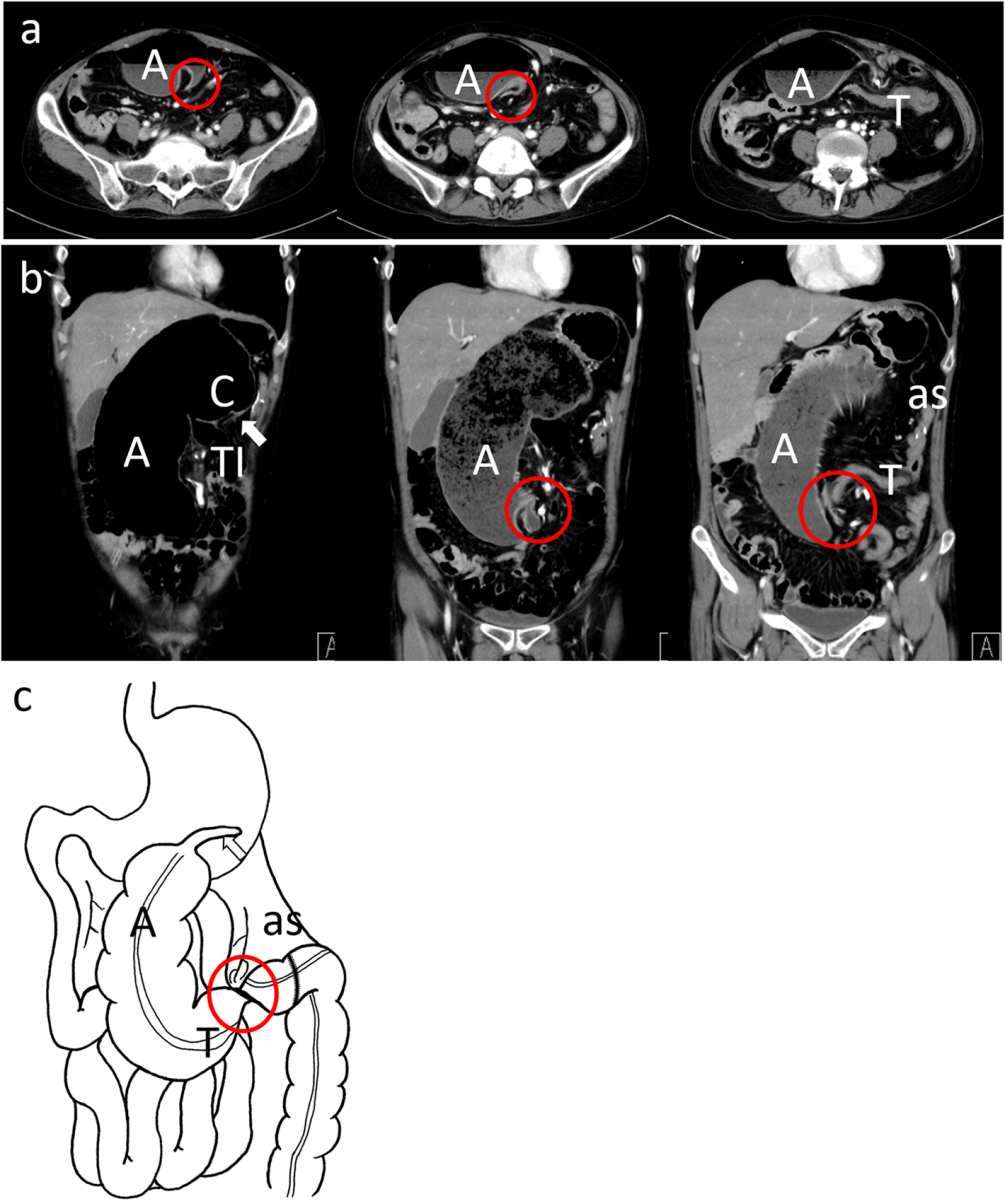
Enhanced computed tomography before the second surgery. Transverse section (**a**) and coronary section (**b**) images show the enlarged cecum (C) and ascending colon (A). The obstructive point was on the remaining transverse colon with a whirlpool sign (red circle). The anastomotic site of the first surgery (as) was placed in the left upper abdominal cavity. The appendix (white arrow) was in the upper abdominal cavity. The volvulus and the distribution of the gastrointestinal tract are indicated in (**c**)

**Fig. 5:**
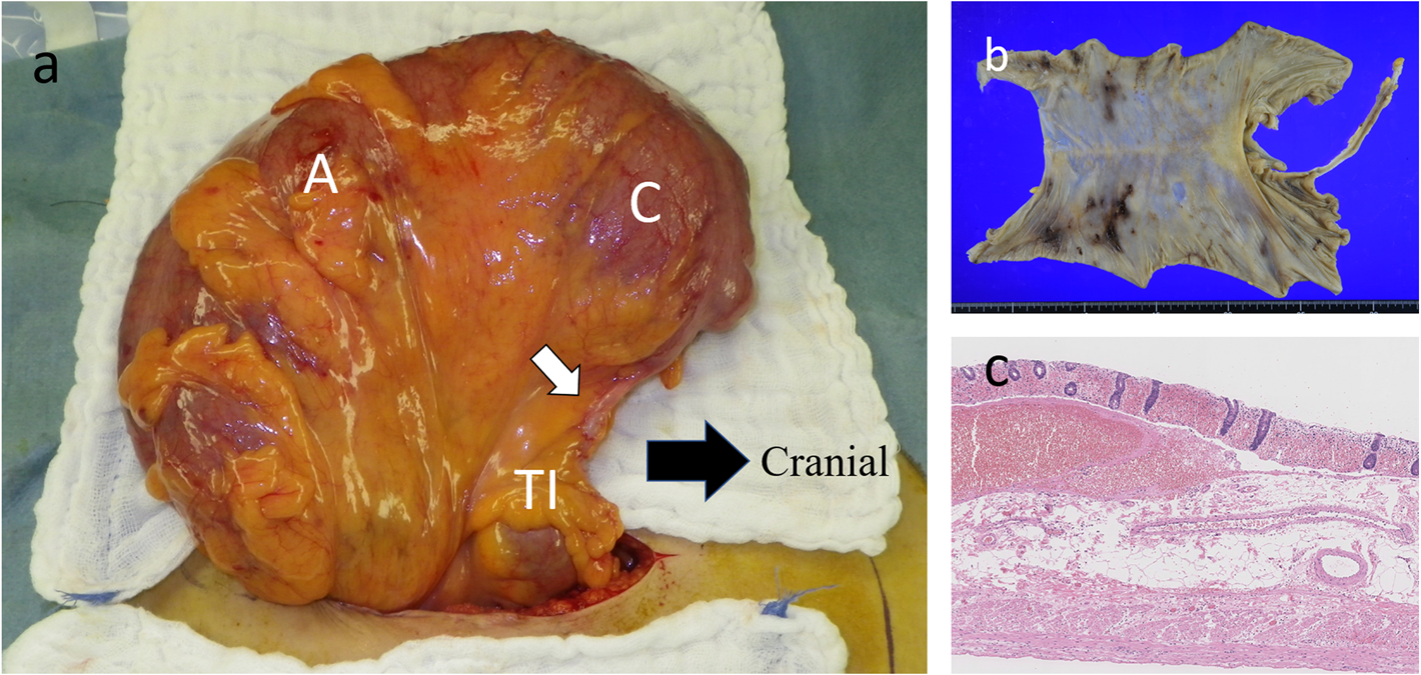
Operative and pathological findings of the second surgery. The operative findings (**a**) showed that the cecum (C) and the appendix (black arrow) were in the upper abdominal cavity, and the ascending colon ran from the cranial to the caudal side. The colon was pulled out from the abdominal cavity without any mobilization. The diagnosis was cecum volvulus with 180° rotation, and the tract was resected from the terminal ileum to the remaining transverse colon (**b**). Pathological findings (**c**) revealed a thinned intestinal wall and edema at the mucosal and submucosal layers, which indicated an acute circulating disturbance

Six years after the first surgery, the patient present to the ER with abdominal fullness and lower abdominal pain. On physical examination, her vital signs were normal. Abdominal examination revealed distension and light lower abdominal tenderness without peritoneal irritation. Her laboratory data were within the normal range. Enhanced CT revealed the dilated colon with an obstruction at the ascending colon (Fig. 4). Although a focal beak sign and narrowing of the vein were noted, there was no sign of ischemia. The appendix was located on the left upper side of the abdominal cavity, unlike in the previous surgery. Clinical diagnosis was volvulus of the cecum and intestinal nonrotation, and emergency laparotomy was performed. In the surgery, the tract from the dilated cecum to the remaining transverse colon was extracted from the abdominal cavity without any resistance (Fig. 5). There was torsion of the terminal ileum to the remaining transverse colon with a 180° clockwise rotation. The dilated and twisted tract was removed, and ileocolic anastomosis with FEEA was performed. The anastomotic site of the previous surgery was adhered and fixed to surrounding tissue. She was discharged uneventfully on postoperative day 9. Although the patient was diagnosed with obstruction of the intestine caused by an operative adhesion 7 months after the surgery, conservative treatment relieved her symptoms in 3 days of hospitalization.

## Discussion

The true incidence of intestinal rotational disorders is unknown, although a report indicates that the ratio is approximately 0.2–1% of the population and that patients present symptomatically at a rate of 1 in 2500 [16]. This condition is the result of an error during the embryonic period. The classification of these anomalies is divided into nonrotation, malrotation, incomplete rotation, paraduodenal hernia, and reverse rotation [5]. Nonrotation is characterized by the duodenum traveling descending straight down to the right side of the SMA and the small intestine occupying the right side of the abdominal cavity and the large intestine located on the left side. Incomplete rotation or malrotation causes duodenal obstruction due to the formation of Ladd’s bands and the lack of duodenal loop rotation. This condition can lead to catastrophic midgut volvulus. Paraduodenal hernia is caused by failure of the 180° counterclockwise rotation of the midgut. The small intestine herniates between the ascending mesocolon and the retroperitoneum. Reversed rotation presents as the transverse colon located inferior to the duodenum and causes partial mesenteric arterial, venous, and lymphatic obstruction.

Abnormal rotation of the intestine tends to be discussed as a pediatric disease; however, adult patients suffer from this condition with either acute or chronic symptoms [15]. Acute symptoms are caused by sudden obstruction and ischemic changes in the intestine, as in our case. Chronic symptoms are not specific, such as abdominal pain, vomiting, and diarrhea, and can last several years [4, 7, 17, 18]. The diagnosis of nonrotation in adults is not easy before the appearance of acute symptoms of obstruction. This is because patients usually develop without any symptoms or with mild chronic symptoms, and the number of cases is too small to identify [7, 19]. Diagnosis is mainly conducted with CT scan, upper gastrointestinal examination, or incidental surgical findings [5]. However, radiographic examination is limited to diagnosing the anomaly of the rotation due to false positives and negatives [17, 20, 21]. Operative findings are vital for the final diagnoses of an abnormality. In our case, the patient showed acute symptoms, and the CT findings suggested the possibility of volvulus and nonrotation [17, 22, 23]. Finally, the operative findings confirmed that she had nonrotation with volvulus.

Colonic volvulus is the condition of bowel torsion around its own mesentery and is the third leading cause of large intestine obstruction [2]. Intestinal volvulus patients tend to have a long redundant colonic segment and elongated mesentery with a narrow base [24, 25]. These anatomical characteristics are either congenital or acquired. One of the congenital causes is the anomaly of intestinal rotation [20, 26, 27]. This anomaly presents as a nonfixed colon and narrowed stalk formation with Ladd’s bands or mesentery adhesions. In our opinion, in cases of adult nonrotation patients, very loose volvulus intermittently occurs with light abdominal symptoms such as pain and vomiting. Loose volvulus and highly mobilized mesentery may cause friction and inflammation at the mesentery. Inflammation leads to the formation of fibrous adhesions at the mesentery and narrows the stalk. Adhesions play a role to serve as an axis in acute severe volvulus at any age. Consequently, once the volvulus attack due to a congenital intestinal rotation anomaly occurs, surgical resection of the involved intestine is the primary indication for radical surgery for treatment and prevention of recurrence. The surgical procedure should be noted along with Ladd’s procedure [28, 29]. First, reduction of the volvulus is required. Second, any fixed band between the cecum or ascending colon and abdominal wall or the duodenum should be dissected to widen the stalk of the mesentery. Third, the adhesion around the duodenum should be detached to mobilize the proximal jejunum to the right upper quadrant. Forth, the involved segment should be removed, and the anastomotic site should be located far from the duodenum to avoid shortening the mesentery stalk, which may lead to further volvulus. Finally, the bowel should be placed in a nonrotation position with appendectomy. There are several reports that indicate a second volvulus attack after Ladd’s procedure in infants for midgut volvulus [30]. This supports our rare case in which an appropriate surgical procedure was performed for volvulus with nonrotation, but volvulus recurrence occurred. Hence, what gastrointestinal surgeons should know is that the intestinal rotation anomaly can suddenly affect adult patients and lead to fatal volvulus, and volvulus recurrence may occur even several years after surgery.

## Conclusions

Volvulus in adulthood due to an anomaly of intestinal rotation is rare and requires surgical treatment. Although a relevant surgical procedure is performed, patients can suffer recurrence as in our case. Hence, surgeons should recognize this condition as not only a pediatric disease but also adult gastrointestinal disorder.

## Data Availability

Data sharing is not applicable to this article, because no datasets were generated or analyzed during this study.

## Abbreviations

CT: Computed tomography
ER: Emergency room
FEEA: Functional end-to-end anastomosis
SMA: Superior mesenteric artery
SMV: Superior mesenteric vein
